# Management of pelvic organ prolapse of ruptured and extruded bladder from a rare complication of vaginal hysterectomy: a case presentation

**DOI:** 10.1186/s12893-021-01047-z

**Published:** 2021-01-19

**Authors:** Piao-Piao Ye, Xiao-Jian Yan, Yasmeen Bano, Hong-Qin Zhao, Feng-Feng Xie, Fang-Yi Zhang, Yu-Feng Wang, Hui Xie, Hai-Hong Jiang

**Affiliations:** 1 Department of Gynecology, The First Affiliated Hospital of Wenzhou Medical University, Zhejiang Province, Wenzhou, China; 2 Center for Uterine Cancer Diagnosis & Therapy Research of Zhejiang Province, Hangzhou, China; 3 Department of Urology, The First Affiliated Hospital of Wenzhou Medical University, Zhejiang Province, Wenzhou, China; 4 Department of Clinical Research, The First Affiliated Hospital of Wenzhou Medical University, Zhejiang Province, Wenzhou, China; 5 Department of Obstetrics, The First Affiliated Hospital of Wenzhou Medical University, Zhejiang Province, Wenzhou, China

**Keywords:** Urinary incontinence, Pelvic organ prolapse, Bladder extrusion, Vaginal hysterectomy, Suprapubic cystostomy

## Abstract

**Background:**

The prolapse of a ruptured and extruded bladder after vaginal hysterectomy is rare in clinical practice. We report the case of a significant mass that prolapsed from the vagina after a vaginal hysterectomy in a multiparous postmenopausal woman.

**Case presentation:**

A 67-year old multiparous postmenopausal Chinese woman was found to have a significant mass extruding from the vagina after a vaginal hysterectomy. The mass was a ruptured and everted bladder, and the diagnosis was confirmed after physical and imaging examinations and urethral catheterization. The patient underwent an emergency operation for mass reduction, bladder repair, and partial colpocleisis under general anesthesia. She recovered without prolapse or urinary drainage complications after 35 months of follow-up.

**Conclusions:**

The present case serves as a guide for the management of patients with pelvic organ prolapse. The condition of patients should be carefully evaluated before surgery, and individualized operation should be performed. Careful postoperative follow-up is crucial for the timely exclusion of complications, especially in elderly patients with persistently increased abdominal pressure.

## Background

The incidence of bladder prolapse, with accompanying rupture and extrusion, is rare in the clinical setting. Few cases have been reported, which have occurred in elderly women with a history of multiple births, malignant diseases, outflow obstruction, trauma, or other combined factors [1, 2]. Pelvic organ prolapse (POP) is one of the most common indications for hysterectomy, especially in postmenopausal women. Studies have reported that 6–8% of adult women had symptomatic POP, which accounted for approximately 14% of all the hysterectomies in the United States [3–6]. Treatment for POP comprises conservative management and surgery. Conservative management includes behavioral modification, pelvic floor muscle exercises, and a mechanical device such as a pessary.

Here, we present a rare case of bladder prolapse, rupture, and extrusion after a vaginal hysterectomy. This report aims to provide some insight regarding the management of POP with regard to bladder rupture and extrusion and to further discuss its related effects and potential preventative methods after vaginal hysterectomy.

## Case presentation

The patient was a 67-year-old woman who was severely malnourished (height: 145 cm, weight: 35 kg, BMI: 14.27 kg/m^2^). She had a 30-year history of POP, 20-year history of chronic bronchitis, and a 5-year history of hypertension. The patient was diagnosed with “uterine prolapse IV and coloptosis III” according to the POP-Q indexing at a local regional hospital. Gynecological examination showed severe prolapse of the anterior and posterior vaginal walls, mucosal thickening of the vaginal wall, surface ulceration, and complete uterine protrusion from the vaginal orifice. The patient had undergone vaginal hysterectomy combined with colporrhaphy for anterior–posterior repair. During the operation, the anterior and posterior fornices were opened along the cervix through the vagina, and the sacral ligament, main ligament, uterine artery and vein, round ligament, and ovarian ligament were cut and gradually sutured. The vaginal stump was sutured with 2–0 sutures. The bulging bladder fascia was sutured 2 times with a purse string, and the anterior vaginal wall was sutured continuously. The redundant posterior vaginal wall was removed and gradually repaired. She experienced urine leakage 1 week after discharge from the hospital; however, she paid no attention to it and did not visit the office.

**Fig. 1:**
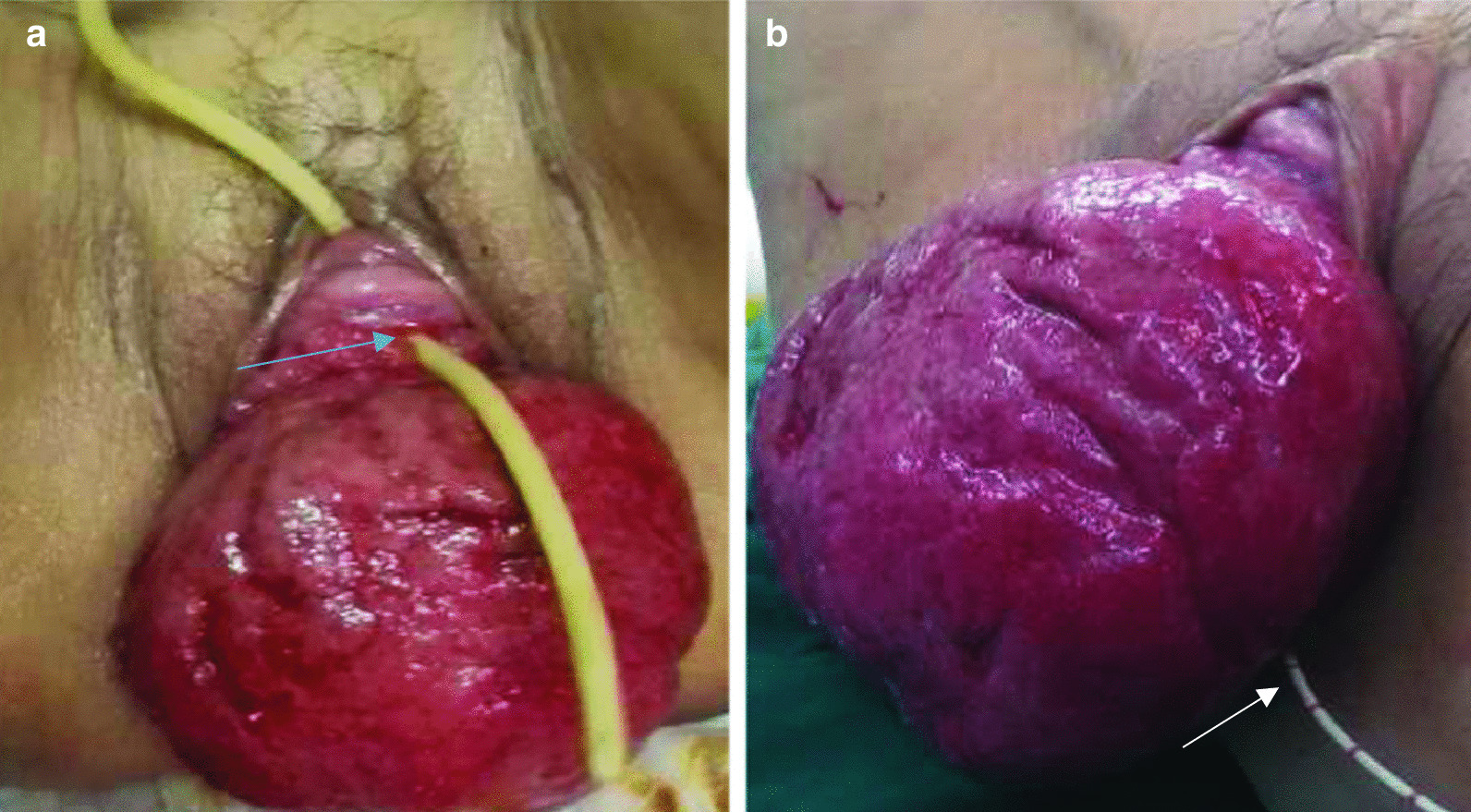
**a** The catheter was passed through the surface of the ruptured and extruded bladder. Blue arrow: Internal urethral meatus. White arrow: Ureteral catheter (left). **b** The ureteral catheter could only be inserted into the left side of the ureter with dribbing urine

It was not until 3 months after the operation that she presented with a mass (size 3 × 3 cm) bulging out of the vagina, in addition to notable coughing from chronic bronchitis. She noted that the vaginal bulge could be pushed back manually at the beginning. However, the mass gradually became larger with continuous coughing and could not be pushed back manually 3 days later, and was accompanied with notable abdominal pain and hematuria. Evenutally, the patient presented to the regional hospital because of a congestive and hyperemic edematous mass (size 10 × 12 × 10 cm) that protruded from the vaginal orifice about 3 months after the operation. The mass had a tough texture with mild bleeding and urine leakage on its surface during the physical examination. The tip of the catheter was passed through the surface of the mass after inserting it into the urethral orifice for about 5 cm (Fig. 1a). An emergency abdominal CT scan showed that the bladder was not visible in the pelvic cavity. She was then referred to our hospital after unsuccessful manual reduction of the mass under epidural anesthesia at a local regional hospital.

She was diagnosed with post-hysterectomy bladder prolapse, rupture, and extrusion after a detailed medical history and physical examination. An emergency exploratory laparotomy was collaboratively performed by gynecologists and urologists after relevant consultations and preoperative tests. The bladder could not be found in the original pelvic location except for the fundus of the bladder. A ureteral catheter was successfully inserted into the ureter (left), while catheter insertion was unsuccessful on the right side of the ureteral orifice because of valgus extrusion and edema of the internal bladder surface (Fig. 1b).

**Fig. 2:**
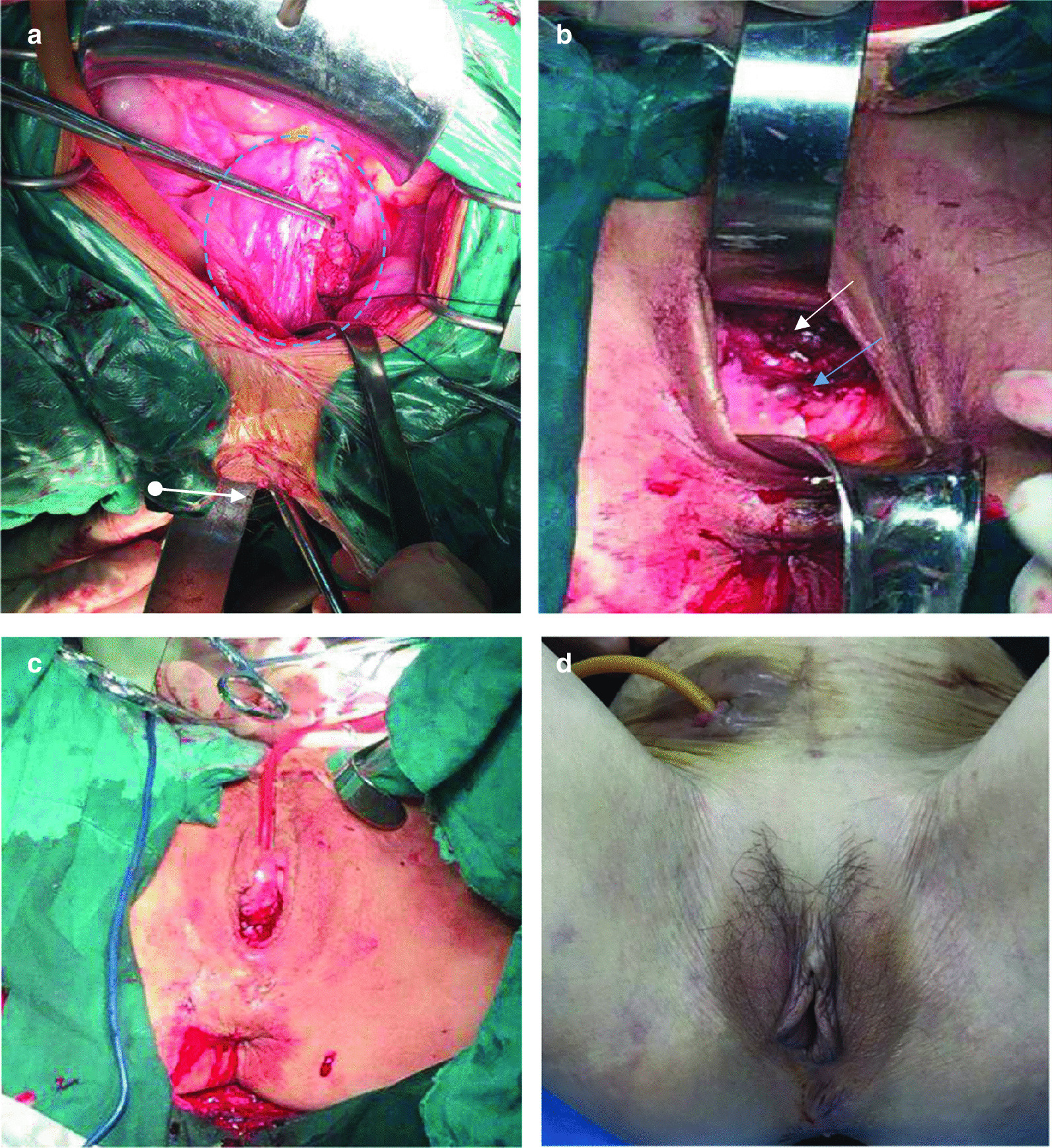
**a** Emergency exploratory laparotomy. Blue circle: Bladder. White arrow: Vagina. **b** Before partial colpocleisis. White arrow: Bladder(repaired). Blue arrow: Anterior wall of vagina. **c** After partial colpocleisis. **d** Follow-up 35 months after surgery

The patient underwent reparation of the bladder after a successful manual reduction of the vaginal bulge. We confirmed that the patient was not involved in any sexual activity. The patient selected the procedure after considering available treatment choices and their advantages and disadvantages. We opted to excise the partial bladder tissue during the reduction and to repair the ruptured bladder after returning it to its anterior pelvic compartment. A suprapubic cystostomy with a small incision was made above the pubic symphysis. The transurethral and transvaginal methylene blue test demonstrated no related urine leakage, indicating the integrity of the bladder wall after the above procedures. Finally, partial colpocleisis was performed to strengthen vaginal support (Fig. 2a–c).

The pelvic drainage tube and vaginal stump were removed 5 days after the procedure, followed by transurethral catheter removal 1 week after the procedure. The patient recovered and was discharged with a suprapubic cystostomy 10 days after the procedure. She maintained the cystostomy with no bulge symptoms for up to 35 months after the procedure (Fig. 2d). The patient had a well-supported vaginal wall in all compartments and no evidence of prolapse according to a pelvic examination during her last office visit. However, the patient complained of being unable to completely void urine. This could have been due to impaired bladder contraction. We have considered closing it to allow her void substantially or to encourage her to perform intermittent self-catheterization.

## Discussion and conclusions

Urinary tract injury is a common complication in pelvic surgeries, accounting for about 0.5–1.5% of the complications in all gynecology-related procedures. Bladder laceration as an acute complication may occur during surgery, while a fistula, which is a chronic complication (0.1–0.4% incidence), usually occurs at a later time. The occurrence of bladder rupture and extrusion in the postoperative period is rare, and over 90% of cases are associated with serious pelvic fractures [2, 7, 8]. To our best knowledge, this is the first report of a patient with the both bladder rupture and extrusion with no pelvic fractures except after a scheduled procedure.

Surgical treatment is most frequently applied in POP, although various conventional surgical procedures have been associated with a high risk of subsequent operations for POP, especially following a vaginal approach, with a reoperation rate of nearly 29% [6]. Hysterectomy is considered an essential part of the surgical management of prolapse worldwide. In the last decade, the importance of hysterectomy in the treatment of prolapse has been increasingly questioned [9, 10]. Some studies have indicated that hysterectomy affects normal supporting structures, which may further induce pelvic floor dysfunction and increase related surgical complications [11].

Surgery for POP aims to restore the vaginal anatomy and its related functions. However, the postoperative risk in women aged above 65 years is up to 13.6-fold higher than in young women [12, 13]. Partial colpocleisis may be used if POP occurs in aging women who have multiple comorbidities and who do not require sexual function. Partial colpocleisis was first described in 1877 by Leon Le Fort [14] as a procedure for vaginal closure. The advantages of partial colpocleisis include a shorter operative time, less blood loss, faster recovery, and simpler anatomical recovery [15]. A recent study involving 47 patients (mean age: older group vs. younger group 84 ± 3.3 vs. 70.8 ± 6.1 years) who underwent Le Fort colpocleisis demonstrated that both objective and subjective cure rates were similar in the two groups, suggesting Le Fort colpocleisis may be an optimal choice for elderly women with POP and serious medical comorbidities [16]. As a result, we performed partial colpocleisis to strengthen vaginal support after the reduction and repair of the ruptured bladder.

The current case highlights several points regarding the clinical experience for POP procedures. First, hysterectomy for POP should be carefully evaluated before the procedure, especially in the presence of associated medical conditions. The patient’s history of chronic bronchitis, which could cause a frequent sudden increase in abdominal pressure, could provoke postoperative POP recurrence. An additional deteriorating factor in the current case was her extremely malnourished state which prolonged the recovery of the vaginal stump. We believe that if patients with chronic bronchitis are managed with standard care, with relief from cough, the possibility of such complications could be effectively decreased. When we inquired about the occurrence of bladder prolapse, the patient claimed she noticed the urinary leakage after the removal of the catheter 1 week after vaginal hysterectomy. At this point, a potential vesicovaginal fistula may have occurred independently or simultaneously. We could not confirm this because of the absence of examination records because the patient was at home. Excessive resection of the anterior vaginal wall could also influence vaginal stump healing, which may cause incision tension. The prolapsed bladder could become enlarged after a partial vaginal stump rupture. Bladder tissue with severe edema and necrosis could ultimately induce bladder rupture. Therefore, regular follow-up after the procedure is very important, especially if the individual has related risk factors affecting recovery after the pelvic reconstruction procedure.

From this case management, we believe that such serious complications may occur after vaginal hysterectomy if a patient has associated risk factors, such as malnutrition and continuous cough. Although surgical skill is important during the management of excessive resection of the anterior vaginal wall, a postoperative office visit for follow-up care is equally important to discover any related complications in advance and to avoid further delay in the healing process.

## Data Availability

Not applicable.

## Abbreviations

POP: Pelvic organ prolapse
